# Crystal structures of two substituted thia­zolidine derivatives

**DOI:** 10.1107/S2056989016011336

**Published:** 2016-07-19

**Authors:** Vijayan Viswanathan, Naga Siva Rao, Raghavachary Raghunathan, Devadasan Velmurugan

**Affiliations:** a Centre of Advanced Study in Crystallography and Biophysics, University of Madras, Guindy Campus, Chennai 600 025, India; b Department of Organic Chemistry, University of Madras, Guindy Campus, Chennai 600 025, India

**Keywords:** crystal structure, thia­zolidine deriv­ative, ferrocen­yl, ace­naphthyl­ene, chromane, hydrogen bonding, C—H⋯π inter­actions

## Abstract

In the first of the compounds reported herein, namely 6′-ferrocenyl-6a′-nitro-6′,6a′,6b′,7′,9′,11a′-hexa­hydro-2*H*-spiro­[ace­naphthyl­ene-1,11′-chromeno[3′,4′:3,4]pyrrolo­[1,2-*c*]thia­zol]-2-one, [Fe(C_5_H_5_)(C_29_H_21_N_2_O_4_S)], (I), the thia­zolidine ring adopts a twist conformation on the methine N—C atoms. In the second compound, *viz.* 6′-(4-methoxy­phen­yl)-6a′-nitro-6′,6a′,6b′,7′,9′,11a′-hexa­hydro-2*H*-spiro­[ace­naphthyl­ene-1,11′-chromeno[3′,4′:3,4]pyrrolo­[1,2-*c*]thia­zol]-2-one, [Fe(C_5_H_5_)(C_26_H_19_N_2_O_5_S)], (II), the thia­zolidine ring adopts an envelope conformation with a methine C atom as the flap. In both compounds, the pyrrolidine ring adopts a twist conformation on the thia­zolidine and tetra­hydro­pyran C atoms. The mean planes of the thia­zolidine and pyrrolidine rings subtend angles of 67.30 (1) and 62.95 (7)° in (I) and (II), respectively, while the mean plane of the pyrrolidine ring makes dihedral angles of 76.53 (1) and 87.74 (7)° with the ace­naphthyl­ene ring system in (I) and (II), respectively. In both compounds, an intra­molecular C—H⋯O hydrogen bond forms an *S*(7) ring motif. In the crystal of (I), mol­ecules are linked *via* two different C—H⋯O hydrogen bonds, forming chains along [001] and [100]. In (II), they are linked through C—H⋯O hydrogen bonds, forming dimers with an *R*_2_^2^(10) ring motif while C—H⋯π inter­actions link the mol­ecules in a head-to-tail fashion, forming chains along the *a*-axis direction.

## Abstract 2

In both compounds, namely 2′-ferrocenyl-6′-methyl-6a’-nitro-6′,6a’,6b’,7′,9′,11a’-hexa­hydro-2*H*-spiro­[ace­naphthyl­ene-1,11′-chromeno[3′,4′:3,4]pyrrolo­[1,2-*c*]thia­zol]-2-one, (I), and 6′-(4-meth­oxy­phen­yl)-6a’-nitro-6′,6a’,6b’,7′,9′,11a’-hexa­hydro-2*H*-spiro­[ace­naphthyl­ene-1,11′-chromeno[3′,4′:3,4]pyrrolo­[1,2-*c*]thia­zol]-2-one, (II), an intra­molecular C—H⋯O hydrogen bond forms an *S*(7) ring motif. In (I), mol­ecules are linked *via* two different C—H⋯O hydrogen bonds, forming chains along [001] and [100]. In (II), they are linked through C—H⋯O hydrogen bonds, forming dimers with an 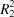
(10) ring motif while C—H⋯π inter­actions link the mol­ecules in a head-to-tail fashion, forming chains along the *a*-axis direction.

## Chemical context   

There are numerous biologically active mol­ecules with five-membered rings containing two hetero atoms. Among them, thia­zolidines are the most extensively investigated class of compounds (Fun *et al.*, 2011 ▸). Thia­zolidine derivatives have attracted continuous inter­est over the years because of their varied biological activities (Shih *et al.*, 2015 ▸). The special importance of the thia­zolidine ring system derives from the fact that it plays an important role in medicinal chemistry. The presence of a thia­zolidine ring in penicillin and related deriv­atives was the first recognition of its occurrence in nature (Čačić *et al.*, 2010 ▸). Substituted thia­zolidine derivatives represent important key inter­mediates for the synthesis of pharmacologically active drugs. The group has wide range of biological activities such as anti­fungal, anti­proliferative, anti-inflammatory, anti­malarial, herbicidal, anti­viral (Samadhiya *et al.*, 2012 ▸), anti­convulsant (Pandey *et al.*, 2011 ▸), anti­cancer and anti-oxidant, and also has inter­esting anti­microbial activity (influenza). In addition, anti­diabetic properties (Majed & Abid, 2015 ▸) have been reported. Thia­zolidine derivatives exhibit anti-HIV, anti­tuberculotic (Fun *et al.*, 2011 ▸), herbicidal, anti­neoplastic, hypolipidemic and anti-inflammatory activities (Vennila *et al.*, 2011 ▸). Thia­zolidines have many inter­esting activity profiles, namely as COX-1 inhibitors, inhibitors of the bacterial enzyme MurB, which is a precursor, acting during the biosynthesis of peptidoglycan, non-nucleoside inhibitors of HIV–RT and anti-histaminic agents (Čačić *et al.*, 2010 ▸).
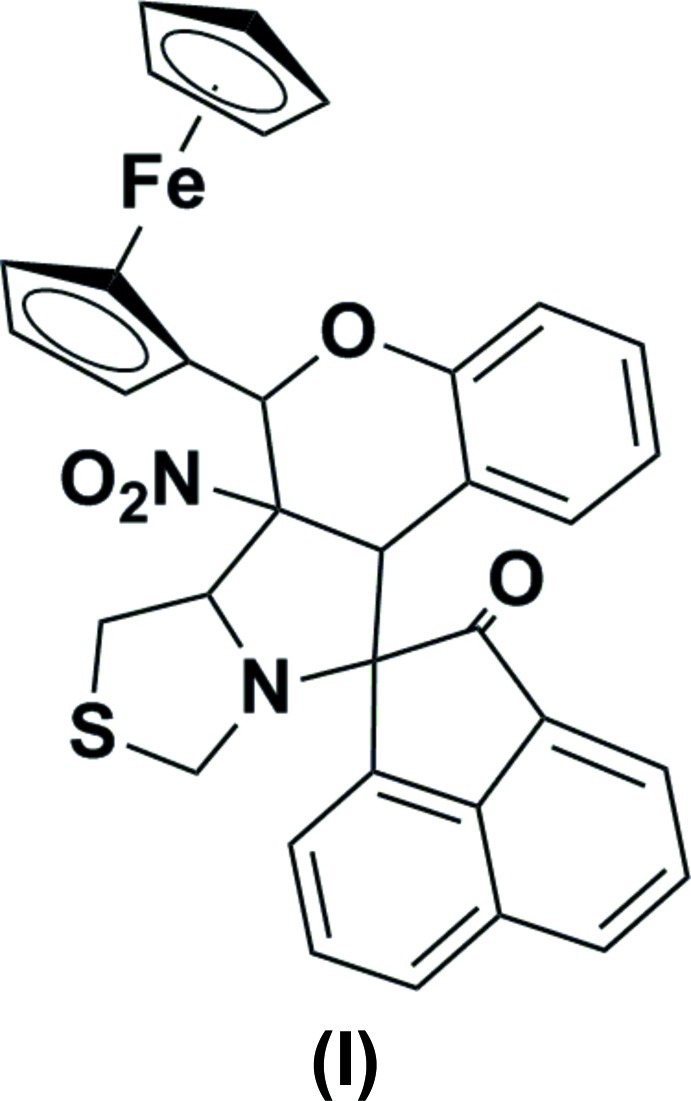
 
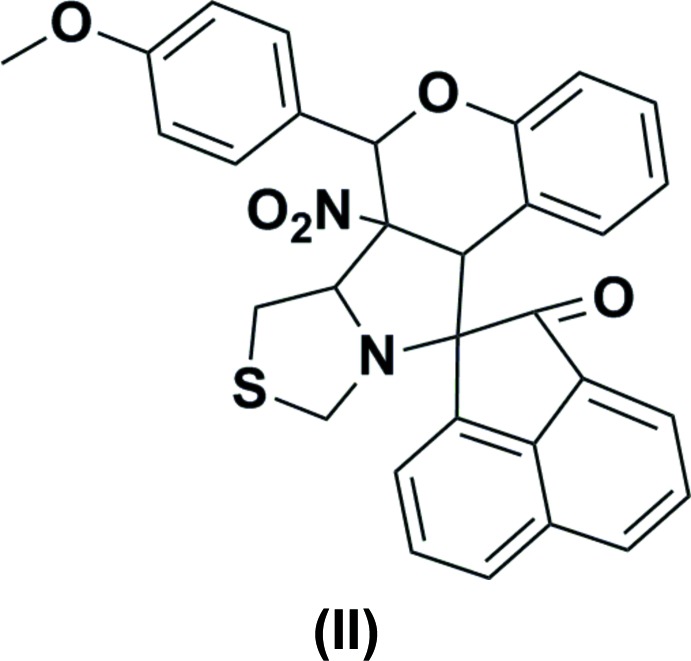


## Structural commentary   

**Figure 1:**
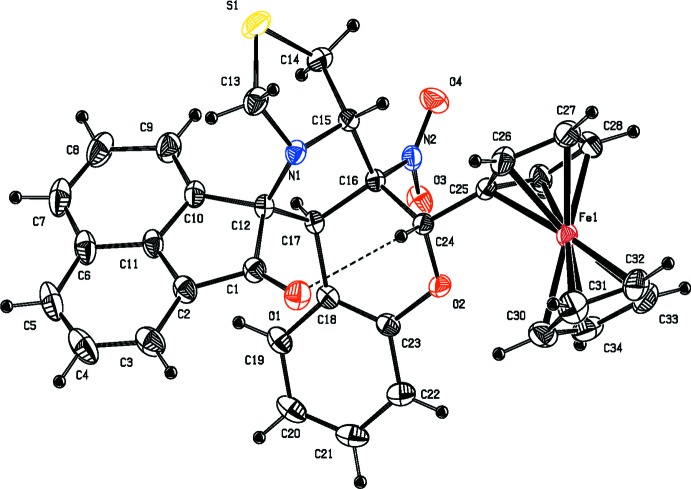
The mol­ecular structure of (I), showing the atom labelling and displacement ellipsoids drawn at 30% probability level. The C—H⋯O contact is shown as a thin dashed line.

**Figure 2:**
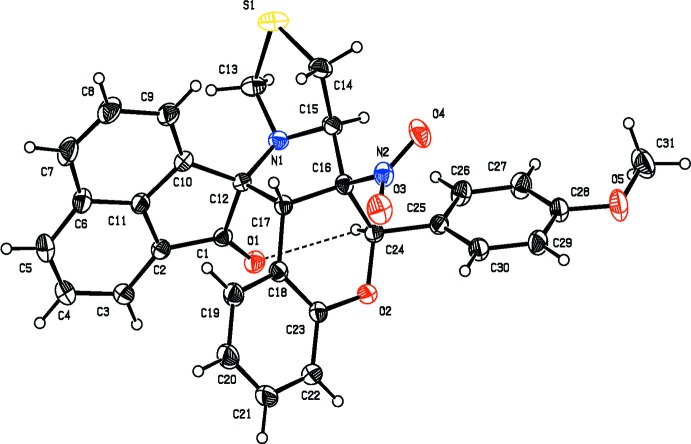
The mol­ecular structure of (II), showing the atom labelling and displacement ellipsoids drawn at 30% probability level. The C—H⋯O contact is shown as a thin dashed line.

In the mol­ecular structures of the compounds reported herein, namely 2′-ferrocenyl-6′-methyl-6a′-nitro-6′,6a′,6b′,7′,9′,11a′-hexa­hydro-2*H*-spiro­[ace­naphthyl­ene-1,11′-chromeno[3′,4′:3,4]pyrrolo­[1,2-*c*]thia­zol]-2-one, (I) (Fig. 1 ▸), and 6′-(4-methoxy­phen­yl)-6a′-nitro-6′,6a′,6b′,7′,9′,11a′-hexa­hydro-2*H*-spiro[ace­naphthyl­ene-1,11′-chromeno[3′,4′:3,4]pyrrolo­[1,2-*c*]thiazol]-2-one, (II) (Fig. 2 ▸), the pyrrolidine ring (C12/N1/C15–C17) is fused with the thia­zolidine ring (N1/C13/S1/C14/C15), the chromane ring system (C16–C23/O2/C24) and the ace­naphthyl­ene ring system (C1–C12). The thia­zolidine ring adopts a twist conformation on the N1—C15 bond with puckering parameters *q*2 = 0.3710 (8) Å, Φ2 = 96.7 (3)° in (I) and an envelope conformation with atom C15 as the flap in (II). The pyrrolidine ring adopts a twist conformation on the C15—C16 bond with puckering parameters *q*2 = 0.3616 (7) Å and Φ2 = 131.3 (3)°, and *q*2 = 0.3829 (8) Å and Φ2 = 123.4 (3)° in the structures of (I) and (II), respectively. The mean planes of the thia­zolidine and pyrrolidine rings are inclined to one another by 67.30 (1) and 62.95 (7)°, while the pyrrolidine and ace­naphthyl­ene ring systems are almost orthogonal to each other [dihedral angles = 76.53 (1) and 87.74 (7)°, respectively]. The chromane ring system adopts a distorted envelope conformation, the flaps being atom C24 in (I), displaced by −0.5585 (1) Å, and atom C16 in (II), displaced by 0.4076 (3) Å.

The pyrrolidine and the chromane ring systems subtend dihedral angles of 74.94 (8) and 67.68 (7)° in (I) and (II), respectively. In (I), the chromane and ferrocene ring systems lie in a plane [C17—C16—C24—C25 = 176.16 (13)° and C23—O2—C24—C25 = −177.50 (13)°]. In (II), the chromane ring system makes a dihedral angle of 62.58 (4)° with the phenyl ring. Atom O1 deviates from the ace­naphthyl­ene ring system by −0.0718 (4) and −0.2218 (3) Å in (I) and (II), respectively.

In both compounds, an intra­molecular C—H⋯O hydrogen bond forms an *S*(7) ring motif (Figs. 1 ▸ and 2 ▸; Tables 1 ▸ and 2 ▸).

## Supra­molecular features   

**Figure 3:**
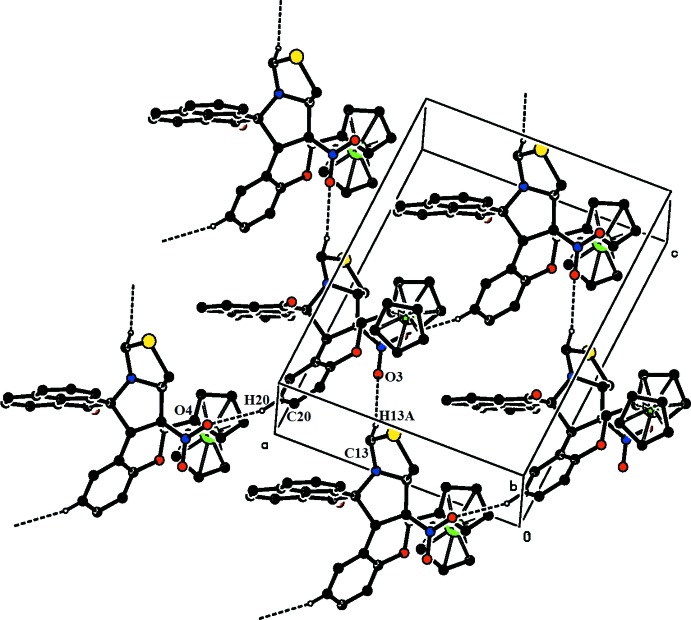
The crystal packing of (I). Note that the C—H⋯O hydrogen bonds (shown as dashed lines) run along [001] and [100] and generate an 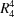
(29) ring motif. H atoms not involved in hydrogen bonds have been excluded for clarity.

**Figure 4:**
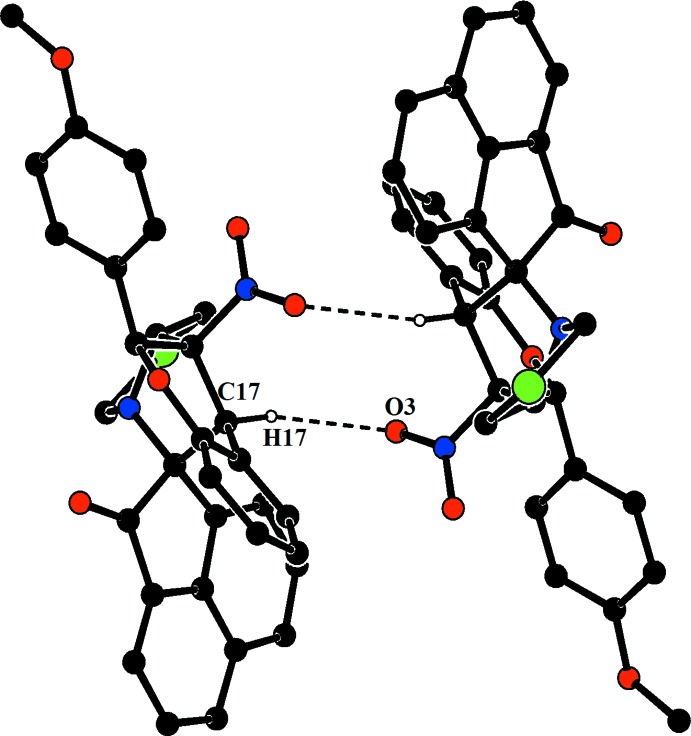
The crystal packing of (II), showing the 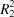
(10) ring motif. H atoms not involved in hydrogen bonds have been excluded for clarity.

**Figure 5:**
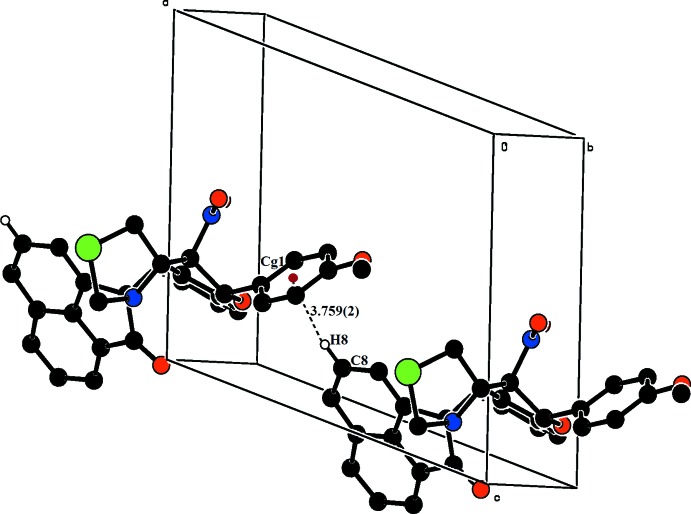
The compound (II) showing the C—H⋯π inter­actions linking mol­ecules in a head-to-tail fashion, forming chains running along the *a* axis. H atoms not involved in hydrogen bonds are omitted for clarity.

In the crystal of (I), mol­ecules are linked *via* C—H⋯O hydrogen bonds along [001] and [100] (Fig. 3 ▸ and Table 1 ▸), generating planes parallel to (010) with embedded 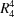
(29) ring motifs. In the crystal of (II), mol­ecules are linked *via* C—H⋯O hydrogen bonds, forming dimers with an 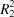
(10) ring motif, as shown in Fig. 4 ▸ and Table 2 ▸. C—H⋯π inter­actions link the mol­ecules in a head-to-tail fashion, forming chains extending along [100] (Fig. 5 ▸).

## Synthesis and crystallization   

Both compounds were obtained through a similar procedure. To a solution of ace­naphtho­quinone (1.0 mmol) and thia­zolidine-4-carb­oxy­lic acid (1.5 mmol) in dry toluene, were added under nitro­gen atmosphere 3-nitro-2-ferrocenyl-2*H*-chromene (1 mmol), for compound (I), or 2-(4-meth­oxy­phen­yl)-3-nitro-2*H*-chromene (1 mmol) for compound (II). The solutions were refluxed for 18 h in a Dean–Stark apparatus to give the corresponding cyclo­adduct. After completion of the reaction as indicated by TLC, the solvent was evaporated under reduced pressure. The crude product obtained was purified by column chromatography using hexa­ne/EtOAc (8:2) as eluent [Yields: 91% for (I), 88% for (II)].

## Refinement   

Crystal data, data collection and structure refinement details are summarized in Table 3 ▸. The hydrogen atoms were placed in calculated positions with C—H = 0.93–0.98 Å and refined using a riding model with fixed isotropic displacement parameters: *U*_iso_(H) = 1.5*U*_eq_(C) for the methyl group and *U*_iso_(H) = 1.2*U*_eq_(C) for the remaining H atoms.

## Supplementary Material

Crystal structure: contains datablock(s) global, I, II. DOI: 10.1107/S2056989016011336/bg2588sup1.cif

Structure factors: contains datablock(s) I. DOI: 10.1107/S2056989016011336/bg2588Isup2.hkl

Structure factors: contains datablock(s) II. DOI: 10.1107/S2056989016011336/bg2588IIsup3.hkl

CCDC references: 1023737, 1023726

Additional supporting information:  crystallographic information; 3D view; checkCIF report

## Figures and Tables

**Table 1:** Hydrogen-bond geometry (Å, °) for (I)

*D*—H⋯*A*	*D*—H	H⋯*A*	*D*⋯*A*	*D*—H⋯*A*
C13—H13*A*⋯O3^i^	0.97	2.50	3.417 (3)	157
C20—H20⋯O4^ii^	0.93	2.59	3.440 (3)	152
C24—H24⋯O1	0.98	2.51	3.301 (3)	138

Symmetry codes: (i) 
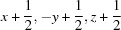
; (ii) 
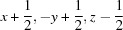
.

**Table 2:** Hydrogen-bond geometry (Å, °) for (II) *Cg*1 and *Cg*2 are the centroids of the C25–C30 and C2–C11 rings, respectively.

*D*—H⋯*A*	*D*—H	H⋯*A*	*D*⋯*A*	*D*—H⋯*A*
C17—H17⋯O3^i^	0.98	2.47	3.412 (2)	161
C24—H24⋯O1	0.98	2.50	3.178 (19)	126
C8—H8⋯*Cg*1^ii^	0.93	2.82	3.759 (2)	148
C27—H27⋯*Cg*2^iii^	0.93	2.79	3.720 (3)	149

Symmetry codes: (i) 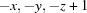; (ii) 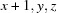; (iii) 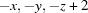.

**Table 3:** Experimental details

	(I)	(II)
Crystal data		
Chemical formula	[Fe(C_5_H_5_)(C_29_H_21_N_2_O_4_S)]	C_31_H_24_N_2_O_5_S
*M*_r_	614.48	536.58
Crystal system, space group	Monoclinic, *P*2_1_/*n*	Triclinic, *P* 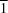
Temperature (K)	293	293
*a*, *b*, *c* (Å)	11.782 (5), 16.741 (5), 14.147 (5)	11.1123 (5), 11.6373 (2), 12.4095 (3)
α, β, γ (°)	90, 98.013 (5), 90	117.812 (1), 110.812 (1), 95.468 (1)
*V* (Å^3^)	2763.1 (17)	1258.89 (7)
*Z*	4	2
Radiation type	Mo *K*α	Mo *K*α
μ (mm^−1^)	0.67	0.18
Crystal size (mm)	0.19 × 0.16 × 0.11	0.22 × 0.18 × 0.10

Data collection		
Diffractometer	Bruker SMART APEXII area-detector	Bruker SMART APEXII area-detector
Absorption correction	Multi-scan (*SADABS*; Bruker, 2008 ▸)	Multi-scan (*SADABS*; Bruker, 2008 ▸)
*T*_min_, *T*_max_	0.746, 0.845	0.746, 0.845
No. of measured, independent and observed [*I* > 2σ(*I*)] reflections	25994, 6900, 5281	18670, 5157, 4192
*R*_int_	0.028	0.023
(sin θ/λ)_max_ (Å^−1^)	0.668	0.626

Refinement		
*R*[*F* ^2^ > 2σ(*F* ^2^)], *wR*(*F* ^2^), *S*	0.035, 0.097, 1.03	0.037, 0.105, 1.04
No. of reflections	6900	5157
No. of parameters	379	353
H-atom treatment	H-atom parameters constrained	H-atom parameters constrained
Δρ_max_, Δρ_min_ (e Å^−3^)	0.29, −0.33	0.25, −0.29

Computer programs: *APEX2* and *SAINT* (Bruker, 2008 ▸), *SHELXS97* (Sheldrick, 2008 ▸), *SHELXL2014* (Sheldrick, 2015 ▸), *ORTEP-3 for Windows* (Farrugia, 2012 ▸) and *PLATON* (Spek, 2009 ▸).

## supplementary crystallographic information

### (I) 6'-Ferrocenyl-6a'-nitro-6',6a',6b',7',9',11a'-hexahydro-2H-spiro[acenaphthylene-1,11'-chromeno[3',4':3,4]pyrrolo[1,2-c]thiazol]-2-one Crystal data

**Table:**

[Fe(C_5_H_5_)(C_29_H_21_N_2_O_4_S)]	*F*(000) = 1272
*M**_r_* = 614.48	*D*_x_ = 1.477 Mg m^−^^3^
Monoclinic, *P*2_1_/*n*	Mo *K*α radiation, λ = 0.71073 Å
*a* = 11.782 (5) Å	Cell parameters from 6900 reflections
*b* = 16.741 (5) Å	θ = 1.9–28.3°
*c* = 14.147 (5) Å	µ = 0.67 mm^−^^1^
β = 98.013 (5)°	*T* = 293 K
*V* = 2763.1 (17) Å^3^	Block, colourless
*Z* = 4	0.19 × 0.16 × 0.11 mm

### (I) 6'-Ferrocenyl-6a'-nitro-6',6a',6b',7',9',11a'-hexahydro-2H-spiro[acenaphthylene-1,11'-chromeno[3',4':3,4]pyrrolo[1,2-c]thiazol]-2-one Data collection

**Table:**

Bruker SMART APEXII area-detector diffractometer	5281 reflections with *I* > 2σ(*I*)
Radiation source: fine-focus sealed tube	*R*_int_ = 0.028
ω and φ scans	θ_max_ = 28.3°, θ_min_ = 1.9°
Absorption correction: multi-scan (*SADABS*; Bruker, 2008)	*h* = −15→12
*T*_min_ = 0.746, *T*_max_ = 0.845	*k* = −22→20
25994 measured reflections	*l* = −16→18
6900 independent reflections	

### (I) 6'-Ferrocenyl-6a'-nitro-6',6a',6b',7',9',11a'-hexahydro-2H-spiro[acenaphthylene-1,11'-chromeno[3',4':3,4]pyrrolo[1,2-c]thiazol]-2-one Refinement

**Table:**

Refinement on *F*^2^	0 restraints
Least-squares matrix: full	Hydrogen site location: inferred from neighbouring sites
*R*[*F*^2^ > 2σ(*F*^2^)] = 0.035	H-atom parameters constrained
*wR*(*F*^2^) = 0.097	*w* = 1/[σ^2^(*F*_o_^2^) + (0.0472*P*)^2^ + 0.6353*P*] where *P* = (*F*_o_^2^ + 2*F*_c_^2^)/3
*S* = 1.03	(Δ/σ)_max_ = 0.001
6900 reflections	Δρ_max_ = 0.29 e Å^−^^3^
379 parameters	Δρ_min_ = −0.33 e Å^−^^3^

### (I) 6'-Ferrocenyl-6a'-nitro-6',6a',6b',7',9',11a'-hexahydro-2H-spiro[acenaphthylene-1,11'-chromeno[3',4':3,4]pyrrolo[1,2-c]thiazol]-2-one Special details

**Table:**

Geometry. All esds (except the esd in the dihedral angle between two l.s. planes) are estimated using the full covariance matrix. The cell esds are taken into account individually in the estimation of esds in distances, angles and torsion angles; correlations between esds in cell parameters are only used when they are defined by crystal symmetry. An approximate (isotropic) treatment of cell esds is used for estimating esds involving l.s. planes.

### (I) 6'-Ferrocenyl-6a'-nitro-6',6a',6b',7',9',11a'-hexahydro-2H-spiro[acenaphthylene-1,11'-chromeno[3',4':3,4]pyrrolo[1,2-c]thiazol]-2-one Fractional atomic coordinates and isotropic or equivalent isotropic displacement parameters (Å^2^)

**Table:**

	*x*	*y*	*z*	*U*_iso_*/*U*_eq_	
C1	0.17168 (14)	0.29715 (10)	0.34129 (12)	0.0382 (4)	
C2	0.27528 (15)	0.26349 (12)	0.30982 (13)	0.0437 (4)	
C3	0.36894 (17)	0.29875 (15)	0.27815 (15)	0.0580 (5)	
H3	0.3751	0.3540	0.2741	0.070*	
C4	0.45406 (18)	0.24843 (19)	0.25235 (17)	0.0714 (7)	
H4	0.5173	0.2713	0.2301	0.086*	
C5	0.44852 (18)	0.16710 (18)	0.25835 (16)	0.0683 (7)	
H5	0.5081	0.1363	0.2412	0.082*	
C6	0.35397 (17)	0.12899 (14)	0.29018 (14)	0.0536 (5)	
C7	0.3351 (2)	0.04678 (15)	0.30007 (16)	0.0660 (6)	
H7	0.3897	0.0103	0.2855	0.079*	
C8	0.2377 (2)	0.02016 (13)	0.33066 (17)	0.0649 (6)	
H8	0.2277	−0.0346	0.3372	0.078*	
C9	0.15032 (19)	0.07272 (11)	0.35320 (15)	0.0528 (5)	
H9	0.0834	0.0526	0.3720	0.063*	
C10	0.16661 (15)	0.15301 (10)	0.34672 (12)	0.0397 (4)	
C11	0.26808 (15)	0.18037 (11)	0.31521 (12)	0.0409 (4)	
C12	0.09371 (13)	0.22504 (9)	0.36526 (11)	0.0327 (3)	
C13	0.13859 (17)	0.19957 (12)	0.54151 (13)	0.0478 (4)	
H13A	0.1502	0.2351	0.5961	0.057*	
H13B	0.2128	0.1863	0.5235	0.057*	
C14	−0.06611 (17)	0.13300 (11)	0.49327 (14)	0.0489 (5)	
H14A	−0.0738	0.1007	0.4358	0.059*	
H14B	−0.1330	0.1248	0.5250	0.059*	
C15	−0.05289 (14)	0.22215 (9)	0.46914 (11)	0.0344 (3)	
H15	−0.0782	0.2554	0.5193	0.041*	
C16	−0.10955 (13)	0.25086 (9)	0.37114 (11)	0.0302 (3)	
C17	−0.02634 (13)	0.22598 (9)	0.30174 (11)	0.0305 (3)	
H17	−0.0450	0.1711	0.2810	0.037*	
C18	−0.03280 (14)	0.27774 (10)	0.21409 (11)	0.0335 (3)	
C19	0.02668 (16)	0.25728 (12)	0.13894 (13)	0.0445 (4)	
H19	0.0699	0.2106	0.1426	0.053*	
C20	0.02230 (19)	0.30546 (14)	0.05904 (14)	0.0562 (5)	
H20	0.0621	0.2911	0.0093	0.067*	
C21	−0.04142 (19)	0.37487 (13)	0.05351 (13)	0.0556 (5)	
H21	−0.0439	0.4075	0.0000	0.067*	
C22	−0.10152 (17)	0.39641 (11)	0.12664 (12)	0.0462 (4)	
H22	−0.1445	0.4432	0.1228	0.055*	
C23	−0.09665 (14)	0.34702 (10)	0.20609 (11)	0.0351 (3)	
C24	−0.12592 (14)	0.34274 (9)	0.36993 (10)	0.0310 (3)	
H24	−0.0518	0.3674	0.3930	0.037*	
C25	−0.21170 (15)	0.37219 (9)	0.43064 (11)	0.0360 (4)	
C26	−0.1857 (2)	0.41094 (13)	0.52068 (13)	0.0546 (5)	
H26	−0.1090	0.4222	0.5545	0.065*	
C27	−0.2915 (2)	0.43003 (14)	0.55249 (15)	0.0691 (7)	
H27	−0.3003	0.4579	0.6119	0.083*	
C28	−0.3806 (2)	0.40483 (13)	0.48430 (18)	0.0621 (6)	
H28	−0.4625	0.4114	0.4878	0.074*	
C29	−0.33282 (16)	0.36859 (11)	0.40861 (16)	0.0476 (4)	
H29	−0.3759	0.3454	0.3508	0.057*	
C30	−0.1999 (2)	0.54692 (11)	0.33192 (16)	0.0600 (6)	
H30	−0.1325	0.5299	0.3037	0.072*	
C31	−0.1986 (2)	0.59003 (12)	0.41768 (18)	0.0629 (6)	
H31	−0.1301	0.6078	0.4598	0.075*	
C32	−0.3126 (2)	0.60257 (11)	0.43236 (16)	0.0586 (6)	
H32	−0.3377	0.6305	0.4867	0.070*	
C33	−0.3842 (2)	0.56757 (12)	0.35621 (16)	0.0595 (5)	
H33	−0.4682	0.5671	0.3477	0.071*	
C34	−0.3144 (2)	0.53302 (12)	0.29394 (14)	0.0591 (6)	
H34	−0.3413	0.5048	0.2343	0.071*	
N1	0.06793 (12)	0.23745 (8)	0.46323 (9)	0.0346 (3)	
N2	−0.22575 (12)	0.21372 (8)	0.34208 (11)	0.0363 (3)	
O1	0.14788 (12)	0.36653 (7)	0.35017 (10)	0.0508 (3)	
O2	−0.16222 (11)	0.37057 (7)	0.27473 (8)	0.0390 (3)	
O3	−0.26393 (11)	0.21596 (8)	0.25751 (9)	0.0492 (3)	
O4	−0.27784 (11)	0.18748 (9)	0.40367 (11)	0.0580 (4)	
S1	0.06376 (5)	0.10735 (3)	0.57181 (4)	0.06071 (16)	
Fe1	−0.27986 (2)	0.48313 (2)	0.42494 (2)	0.03501 (8)	

### (I) 6'-Ferrocenyl-6a'-nitro-6',6a',6b',7',9',11a'-hexahydro-2H-spiro[acenaphthylene-1,11'-chromeno[3',4':3,4]pyrrolo[1,2-c]thiazol]-2-one Atomic displacement parameters (Å^2^)

**Table:**

	*U*^11^	*U*^22^	*U*^33^	*U*^12^	*U*^13^	*U*^23^
C1	0.0313 (9)	0.0423 (9)	0.0401 (9)	−0.0019 (7)	0.0014 (7)	−0.0009 (7)
C2	0.0288 (9)	0.0600 (11)	0.0413 (9)	−0.0012 (8)	0.0016 (7)	−0.0017 (8)
C3	0.0392 (11)	0.0821 (15)	0.0526 (12)	−0.0127 (10)	0.0066 (9)	−0.0002 (10)
C4	0.0352 (11)	0.125 (2)	0.0563 (13)	−0.0084 (13)	0.0130 (10)	−0.0133 (14)
C5	0.0345 (11)	0.117 (2)	0.0537 (13)	0.0167 (12)	0.0063 (9)	−0.0229 (13)
C6	0.0384 (10)	0.0803 (15)	0.0400 (10)	0.0192 (10)	−0.0023 (8)	−0.0148 (9)
C7	0.0633 (15)	0.0755 (15)	0.0566 (13)	0.0355 (12)	−0.0007 (11)	−0.0184 (11)
C8	0.0830 (17)	0.0436 (11)	0.0657 (14)	0.0229 (11)	0.0015 (13)	−0.0096 (9)
C9	0.0585 (13)	0.0423 (10)	0.0584 (12)	0.0086 (9)	0.0105 (10)	−0.0052 (9)
C10	0.0375 (9)	0.0413 (9)	0.0400 (9)	0.0090 (7)	0.0044 (7)	−0.0042 (7)
C11	0.0312 (9)	0.0562 (11)	0.0340 (9)	0.0100 (8)	−0.0003 (7)	−0.0066 (7)
C12	0.0286 (8)	0.0324 (8)	0.0369 (8)	0.0029 (6)	0.0038 (6)	−0.0023 (6)
C13	0.0474 (11)	0.0517 (11)	0.0411 (10)	0.0094 (8)	−0.0045 (8)	0.0007 (8)
C14	0.0484 (11)	0.0448 (10)	0.0539 (11)	0.0039 (8)	0.0082 (9)	0.0155 (8)
C15	0.0358 (9)	0.0373 (8)	0.0307 (8)	0.0051 (7)	0.0072 (7)	0.0016 (6)
C16	0.0268 (8)	0.0320 (8)	0.0318 (8)	0.0013 (6)	0.0039 (6)	−0.0004 (6)
C17	0.0284 (8)	0.0312 (7)	0.0323 (8)	0.0007 (6)	0.0060 (6)	−0.0034 (6)
C18	0.0308 (8)	0.0406 (8)	0.0290 (8)	−0.0047 (7)	0.0039 (6)	−0.0044 (6)
C19	0.0424 (10)	0.0546 (11)	0.0384 (9)	−0.0024 (8)	0.0120 (8)	−0.0084 (8)
C20	0.0576 (13)	0.0762 (14)	0.0389 (10)	−0.0099 (11)	0.0215 (9)	−0.0057 (9)
C21	0.0658 (14)	0.0681 (13)	0.0338 (9)	−0.0130 (11)	0.0101 (9)	0.0091 (9)
C22	0.0532 (12)	0.0482 (10)	0.0364 (9)	−0.0053 (8)	0.0029 (8)	0.0054 (7)
C23	0.0352 (9)	0.0418 (9)	0.0284 (8)	−0.0051 (7)	0.0045 (6)	−0.0022 (6)
C24	0.0328 (8)	0.0317 (8)	0.0285 (7)	0.0022 (6)	0.0045 (6)	0.0005 (6)
C25	0.0406 (9)	0.0341 (8)	0.0341 (8)	0.0090 (7)	0.0083 (7)	0.0049 (6)
C26	0.0668 (13)	0.0656 (12)	0.0300 (9)	0.0278 (10)	0.0021 (9)	0.0005 (8)
C27	0.100 (2)	0.0739 (15)	0.0400 (11)	0.0418 (14)	0.0312 (13)	0.0130 (10)
C28	0.0616 (14)	0.0558 (12)	0.0778 (16)	0.0136 (10)	0.0413 (13)	0.0157 (11)
C29	0.0403 (10)	0.0371 (9)	0.0687 (12)	−0.0014 (8)	0.0191 (9)	−0.0013 (8)
C30	0.0800 (16)	0.0359 (10)	0.0717 (14)	−0.0013 (10)	0.0380 (13)	0.0044 (9)
C31	0.0682 (15)	0.0406 (10)	0.0811 (16)	−0.0106 (10)	0.0151 (12)	−0.0122 (10)
C32	0.0757 (15)	0.0358 (10)	0.0663 (14)	0.0118 (9)	0.0172 (12)	−0.0083 (9)
C33	0.0657 (14)	0.0489 (11)	0.0620 (13)	0.0194 (10)	0.0019 (11)	0.0094 (10)
C34	0.0931 (18)	0.0450 (11)	0.0387 (10)	0.0105 (11)	0.0074 (11)	0.0085 (8)
N1	0.0331 (7)	0.0372 (7)	0.0323 (7)	0.0056 (6)	0.0005 (6)	−0.0015 (5)
N2	0.0288 (7)	0.0346 (7)	0.0460 (8)	0.0028 (6)	0.0071 (6)	−0.0001 (6)
O1	0.0481 (8)	0.0373 (7)	0.0668 (9)	−0.0048 (6)	0.0071 (7)	−0.0017 (6)
O2	0.0476 (7)	0.0409 (6)	0.0292 (6)	0.0123 (5)	0.0078 (5)	0.0049 (5)
O3	0.0339 (7)	0.0675 (9)	0.0444 (7)	−0.0035 (6)	−0.0003 (6)	−0.0090 (6)
O4	0.0411 (8)	0.0710 (9)	0.0645 (9)	−0.0105 (7)	0.0165 (7)	0.0170 (7)
S1	0.0694 (4)	0.0521 (3)	0.0579 (3)	0.0147 (3)	−0.0006 (3)	0.0201 (2)
Fe1	0.03822 (15)	0.03383 (13)	0.03325 (13)	0.00572 (10)	0.00597 (10)	−0.00222 (9)

### (I) 6'-Ferrocenyl-6a'-nitro-6',6a',6b',7',9',11a'-hexahydro-2H-spiro[acenaphthylene-1,11'-chromeno[3',4':3,4]pyrrolo[1,2-c]thiazol]-2-one Geometric parameters (Å, º)

**Table:**

C1—O1	1.205 (2)	C19—H19	0.9300
C1—C2	1.469 (2)	C20—C21	1.380 (3)
C1—C12	1.582 (2)	C20—H20	0.9300
C2—C3	1.380 (3)	C21—C22	1.380 (3)
C2—C11	1.397 (3)	C21—H21	0.9300
C3—C4	1.397 (3)	C22—C23	1.390 (2)
C3—H3	0.9300	C22—H22	0.9300
C4—C5	1.366 (4)	C23—O2	1.3799 (19)
C4—H4	0.9300	C24—O2	1.4330 (19)
C5—C6	1.411 (3)	C24—C25	1.498 (2)
C5—H5	0.9300	C24—H24	0.9800
C6—C7	1.404 (3)	C25—C29	1.419 (3)
C6—C11	1.410 (2)	C25—C26	1.424 (3)
C7—C8	1.357 (4)	C25—Fe1	2.0206 (16)
C7—H7	0.9300	C26—C27	1.419 (3)
C8—C9	1.424 (3)	C26—Fe1	2.0263 (19)
C8—H8	0.9300	C26—H26	0.9800
C9—C10	1.363 (3)	C27—C28	1.388 (4)
C9—H9	0.9300	C27—Fe1	2.033 (2)
C10—C11	1.409 (3)	C27—H27	0.9800
C10—C12	1.524 (2)	C28—C29	1.414 (3)
C12—N1	1.474 (2)	C28—Fe1	2.027 (2)
C12—C17	1.566 (2)	C28—H28	0.9800
C13—N1	1.437 (2)	C29—Fe1	2.0197 (19)
C13—S1	1.857 (2)	C29—H29	0.9800
C13—H13A	0.9700	C30—C34	1.401 (3)
C13—H13B	0.9700	C30—C31	1.410 (3)
C14—C15	1.544 (2)	C30—Fe1	2.026 (2)
C14—S1	1.813 (2)	C30—H30	0.9800
C14—H14A	0.9700	C31—C32	1.403 (3)
C14—H14B	0.9700	C31—Fe1	2.039 (2)
C15—N1	1.460 (2)	C31—H31	0.9800
C15—C16	1.530 (2)	C32—C33	1.401 (3)
C15—H15	0.9800	C32—Fe1	2.042 (2)
C16—N2	1.508 (2)	C32—H32	0.9800
C16—C17	1.538 (2)	C33—C34	1.410 (3)
C16—C24	1.550 (2)	C33—Fe1	2.030 (2)
C17—C18	1.506 (2)	C33—H33	0.9800
C17—H17	0.9800	C34—Fe1	2.022 (2)
C18—C23	1.378 (2)	C34—H34	0.9800
C18—C19	1.395 (2)	N2—O4	1.2158 (19)
C19—C20	1.384 (3)	N2—O3	1.2186 (19)
			
O1—C1—C2	128.05 (16)	C25—C26—H26	126.3
O1—C1—C12	124.22 (15)	Fe1—C26—H26	126.3
C2—C1—C12	107.71 (14)	C28—C27—C26	108.86 (19)
C3—C2—C11	120.08 (18)	C28—C27—Fe1	69.77 (12)
C3—C2—C1	132.09 (19)	C26—C27—Fe1	69.27 (11)
C11—C2—C1	107.83 (15)	C28—C27—H27	125.6
C2—C3—C4	117.6 (2)	C26—C27—H27	125.6
C2—C3—H3	121.2	Fe1—C27—H27	125.6
C4—C3—H3	121.2	C27—C28—C29	108.34 (19)
C5—C4—C3	122.9 (2)	C27—C28—Fe1	70.25 (13)
C5—C4—H4	118.6	C29—C28—Fe1	69.27 (11)
C3—C4—H4	118.6	C27—C28—H28	125.8
C4—C5—C6	121.1 (2)	C29—C28—H28	125.8
C4—C5—H5	119.4	Fe1—C28—H28	125.8
C6—C5—H5	119.4	C28—C29—C25	108.0 (2)
C7—C6—C11	116.3 (2)	C28—C29—Fe1	69.84 (12)
C7—C6—C5	128.2 (2)	C25—C29—Fe1	69.47 (10)
C11—C6—C5	115.5 (2)	C28—C29—H29	126.0
C8—C7—C6	120.43 (19)	C25—C29—H29	126.0
C8—C7—H7	119.8	Fe1—C29—H29	126.0
C6—C7—H7	119.8	C34—C30—C31	108.0 (2)
C7—C8—C9	122.6 (2)	C34—C30—Fe1	69.59 (12)
C7—C8—H8	118.7	C31—C30—Fe1	70.19 (12)
C9—C8—H8	118.7	C34—C30—H30	126.0
C10—C9—C8	118.8 (2)	C31—C30—H30	126.0
C10—C9—H9	120.6	Fe1—C30—H30	126.0
C8—C9—H9	120.6	C32—C31—C30	108.0 (2)
C9—C10—C11	118.39 (16)	C32—C31—Fe1	70.00 (12)
C9—C10—C12	132.91 (17)	C30—C31—Fe1	69.22 (11)
C11—C10—C12	108.70 (15)	C32—C31—H31	126.0
C2—C11—C10	113.68 (15)	C30—C31—H31	126.0
C2—C11—C6	122.87 (18)	Fe1—C31—H31	126.0
C10—C11—C6	123.45 (19)	C33—C32—C31	108.0 (2)
N1—C12—C10	117.90 (13)	C33—C32—Fe1	69.43 (11)
N1—C12—C17	104.47 (12)	C31—C32—Fe1	69.79 (11)
C10—C12—C17	113.24 (13)	C33—C32—H32	126.0
N1—C12—C1	107.21 (13)	C31—C32—H32	126.0
C10—C12—C1	102.06 (13)	Fe1—C32—H32	126.0
C17—C12—C1	111.98 (13)	C32—C33—C34	108.1 (2)
N1—C13—S1	107.58 (13)	C32—C33—Fe1	70.32 (12)
N1—C13—H13A	110.2	C34—C33—Fe1	69.32 (12)
S1—C13—H13A	110.2	C32—C33—H33	125.9
N1—C13—H13B	110.2	C34—C33—H33	125.9
S1—C13—H13B	110.2	Fe1—C33—H33	125.9
H13A—C13—H13B	108.5	C30—C34—C33	107.9 (2)
C15—C14—S1	105.19 (13)	C30—C34—Fe1	69.93 (12)
C15—C14—H14A	110.7	C33—C34—Fe1	69.95 (12)
S1—C14—H14A	110.7	C30—C34—H34	126.1
C15—C14—H14B	110.7	C33—C34—H34	126.1
S1—C14—H14B	110.7	Fe1—C34—H34	126.1
H14A—C14—H14B	108.8	C13—N1—C15	110.07 (14)
N1—C15—C16	101.46 (12)	C13—N1—C12	119.29 (14)
N1—C15—C14	108.15 (13)	C15—N1—C12	111.06 (13)
C16—C15—C14	117.35 (14)	O4—N2—O3	124.10 (15)
N1—C15—H15	109.8	O4—N2—C16	118.91 (14)
C16—C15—H15	109.8	O3—N2—C16	116.87 (13)
C14—C15—H15	109.8	C23—O2—C24	116.45 (12)
N2—C16—C15	112.49 (13)	C14—S1—C13	92.80 (9)
N2—C16—C17	110.46 (12)	C29—Fe1—C25	41.12 (8)
C15—C16—C17	104.88 (12)	C29—Fe1—C34	105.54 (9)
N2—C16—C24	107.42 (12)	C25—Fe1—C34	116.39 (8)
C15—C16—C24	111.08 (12)	C29—Fe1—C30	126.18 (8)
C17—C16—C24	110.55 (12)	C25—Fe1—C30	106.99 (8)
C18—C17—C16	113.96 (13)	C34—Fe1—C30	40.48 (10)
C18—C17—C12	114.58 (13)	C29—Fe1—C26	69.01 (9)
C16—C17—C12	103.97 (12)	C25—Fe1—C26	41.21 (7)
C18—C17—H17	108.0	C34—Fe1—C26	151.72 (9)
C16—C17—H17	108.0	C30—Fe1—C26	119.19 (10)
C12—C17—H17	108.0	C29—Fe1—C28	40.89 (8)
C23—C18—C19	118.06 (16)	C25—Fe1—C28	68.99 (8)
C23—C18—C17	121.07 (14)	C34—Fe1—C28	126.31 (11)
C19—C18—C17	120.87 (15)	C30—Fe1—C28	164.13 (11)
C20—C19—C18	120.93 (19)	C26—Fe1—C28	68.58 (10)
C20—C19—H19	119.5	C29—Fe1—C33	116.71 (9)
C18—C19—H19	119.5	C25—Fe1—C33	150.45 (8)
C21—C20—C19	119.68 (17)	C34—Fe1—C33	40.73 (9)
C21—C20—H20	120.2	C30—Fe1—C33	68.12 (10)
C19—C20—H20	120.2	C26—Fe1—C33	166.86 (8)
C20—C21—C22	120.62 (18)	C28—Fe1—C33	107.36 (10)
C20—C21—H21	119.7	C29—Fe1—C27	68.18 (10)
C22—C21—H21	119.7	C25—Fe1—C27	68.80 (7)
C21—C22—C23	118.88 (19)	C34—Fe1—C27	164.63 (11)
C21—C22—H22	120.6	C30—Fe1—C27	154.40 (12)
C23—C22—H22	120.6	C26—Fe1—C27	40.93 (9)
C18—C23—O2	122.36 (14)	C28—Fe1—C27	39.98 (10)
C18—C23—C22	121.83 (16)	C33—Fe1—C27	128.18 (9)
O2—C23—C22	115.78 (15)	C29—Fe1—C31	165.30 (8)
O2—C24—C25	107.16 (13)	C25—Fe1—C31	128.46 (9)
O2—C24—C16	110.69 (12)	C34—Fe1—C31	68.11 (10)
C25—C24—C16	114.40 (13)	C30—Fe1—C31	40.58 (9)
O2—C24—H24	108.1	C26—Fe1—C31	109.88 (10)
C25—C24—H24	108.1	C28—Fe1—C31	153.45 (9)
C16—C24—H24	108.1	C33—Fe1—C31	67.79 (10)
C29—C25—C26	107.44 (16)	C27—Fe1—C31	121.25 (11)
C29—C25—C24	126.76 (16)	C29—Fe1—C32	151.41 (9)
C26—C25—C24	125.79 (17)	C25—Fe1—C32	167.14 (9)
C29—C25—Fe1	69.41 (10)	C34—Fe1—C32	68.12 (9)
C26—C25—Fe1	69.61 (10)	C30—Fe1—C32	68.01 (9)
C24—C25—Fe1	125.15 (11)	C26—Fe1—C32	129.74 (9)
C27—C26—C25	107.3 (2)	C28—Fe1—C32	119.10 (9)
C27—C26—Fe1	69.80 (12)	C33—Fe1—C32	40.25 (9)
C25—C26—Fe1	69.18 (10)	C27—Fe1—C32	110.24 (9)
C27—C26—H26	126.3	C31—Fe1—C32	40.22 (9)
			
O1—C1—C2—C3	−3.5 (3)	C21—C22—C23—C18	0.6 (3)
C12—C1—C2—C3	177.9 (2)	C21—C22—C23—O2	−177.60 (17)
O1—C1—C2—C11	177.00 (18)	N2—C16—C24—O2	−65.60 (15)
C12—C1—C2—C11	−1.61 (19)	C15—C16—C24—O2	171.00 (12)
C11—C2—C3—C4	0.0 (3)	C17—C16—C24—O2	54.99 (17)
C1—C2—C3—C4	−179.4 (2)	N2—C16—C24—C25	55.58 (17)
C2—C3—C4—C5	−0.8 (3)	C15—C16—C24—C25	−67.83 (18)
C3—C4—C5—C6	1.0 (4)	C17—C16—C24—C25	176.16 (13)
C4—C5—C6—C7	179.8 (2)	O2—C24—C25—C29	46.3 (2)
C4—C5—C6—C11	−0.3 (3)	C16—C24—C25—C29	−76.8 (2)
C11—C6—C7—C8	1.1 (3)	O2—C24—C25—C26	−132.02 (17)
C5—C6—C7—C8	−179.0 (2)	C16—C24—C25—C26	104.88 (19)
C6—C7—C8—C9	0.6 (4)	O2—C24—C25—Fe1	−43.03 (18)
C7—C8—C9—C10	−2.1 (3)	C16—C24—C25—Fe1	−166.13 (11)
C8—C9—C10—C11	1.8 (3)	C29—C25—C26—C27	0.3 (2)
C8—C9—C10—C12	−179.70 (19)	C24—C25—C26—C27	178.93 (16)
C3—C2—C11—C10	−178.66 (17)	Fe1—C25—C26—C27	59.64 (14)
C1—C2—C11—C10	0.9 (2)	C29—C25—C26—Fe1	−59.33 (12)
C3—C2—C11—C6	0.6 (3)	C24—C25—C26—Fe1	119.29 (16)
C1—C2—C11—C6	−179.89 (16)	C25—C26—C27—C28	−0.6 (2)
C9—C10—C11—C2	179.08 (17)	Fe1—C26—C27—C28	58.67 (15)
C12—C10—C11—C2	0.3 (2)	C25—C26—C27—Fe1	−59.25 (13)
C9—C10—C11—C6	−0.1 (3)	C26—C27—C28—C29	0.6 (2)
C12—C10—C11—C6	−178.96 (16)	Fe1—C27—C28—C29	58.98 (14)
C7—C6—C11—C2	179.51 (18)	C26—C27—C28—Fe1	−58.37 (15)
C5—C6—C11—C2	−0.4 (3)	C27—C28—C29—C25	−0.4 (2)
C7—C6—C11—C10	−1.3 (3)	Fe1—C28—C29—C25	59.17 (12)
C5—C6—C11—C10	178.75 (18)	C27—C28—C29—Fe1	−59.59 (15)
C9—C10—C12—N1	63.2 (3)	C26—C25—C29—C28	0.1 (2)
C11—C10—C12—N1	−118.28 (16)	C24—C25—C29—C28	−178.55 (16)
C9—C10—C12—C17	−59.2 (3)	Fe1—C25—C29—C28	−59.40 (13)
C11—C10—C12—C17	119.36 (15)	C26—C25—C29—Fe1	59.46 (13)
C9—C10—C12—C1	−179.8 (2)	C24—C25—C29—Fe1	−119.15 (16)
C11—C10—C12—C1	−1.18 (17)	C34—C30—C31—C32	0.0 (2)
O1—C1—C12—N1	−52.4 (2)	Fe1—C30—C31—C32	−59.50 (15)
C2—C1—C12—N1	126.23 (14)	C34—C30—C31—Fe1	59.54 (14)
O1—C1—C12—C10	−177.00 (17)	C30—C31—C32—C33	−0.1 (2)
C2—C1—C12—C10	1.68 (17)	Fe1—C31—C32—C33	−59.08 (15)
O1—C1—C12—C17	61.6 (2)	C30—C31—C32—Fe1	59.01 (15)
C2—C1—C12—C17	−119.74 (15)	C31—C32—C33—C34	0.1 (2)
S1—C14—C15—N1	34.99 (16)	Fe1—C32—C33—C34	−59.23 (14)
S1—C14—C15—C16	148.88 (12)	C31—C32—C33—Fe1	59.30 (15)
N1—C15—C16—N2	157.43 (12)	C31—C30—C34—C33	0.0 (2)
C14—C15—C16—N2	39.87 (19)	Fe1—C30—C34—C33	59.93 (14)
N1—C15—C16—C17	37.33 (15)	C31—C30—C34—Fe1	−59.92 (15)
C14—C15—C16—C17	−80.23 (17)	C32—C33—C34—C30	−0.1 (2)
N1—C15—C16—C24	−82.12 (14)	Fe1—C33—C34—C30	−59.91 (14)
C14—C15—C16—C24	160.32 (14)	C32—C33—C34—Fe1	59.86 (15)
N2—C16—C17—C18	85.38 (16)	S1—C13—N1—C15	31.86 (16)
C15—C16—C17—C18	−153.19 (13)	S1—C13—N1—C12	−98.21 (16)
C24—C16—C17—C18	−33.38 (18)	C16—C15—N1—C13	−168.36 (13)
N2—C16—C17—C12	−149.19 (12)	C14—C15—N1—C13	−44.33 (18)
C15—C16—C17—C12	−27.75 (15)	C16—C15—N1—C12	−34.03 (15)
C24—C16—C17—C12	92.05 (14)	C14—C15—N1—C12	90.01 (16)
N1—C12—C17—C18	132.57 (13)	C10—C12—N1—C13	19.8 (2)
C10—C12—C17—C18	−97.87 (16)	C17—C12—N1—C13	146.48 (15)
C1—C12—C17—C18	16.87 (18)	C1—C12—N1—C13	−94.53 (17)
N1—C12—C17—C16	7.54 (15)	C10—C12—N1—C15	−109.86 (16)
C10—C12—C17—C16	137.10 (14)	C17—C12—N1—C15	16.86 (16)
C1—C12—C17—C16	−108.16 (14)	C1—C12—N1—C15	135.85 (13)
C16—C17—C18—C23	8.6 (2)	C15—C16—N2—O4	22.38 (19)
C12—C17—C18—C23	−111.04 (17)	C17—C16—N2—O4	139.20 (15)
C16—C17—C18—C19	−172.07 (15)	C24—C16—N2—O4	−100.16 (16)
C12—C17—C18—C19	68.33 (19)	C15—C16—N2—O3	−161.40 (13)
C23—C18—C19—C20	0.4 (3)	C17—C16—N2—O3	−44.58 (18)
C17—C18—C19—C20	−178.95 (17)	C24—C16—N2—O3	76.06 (16)
C18—C19—C20—C21	0.2 (3)	C18—C23—O2—C24	26.5 (2)
C19—C20—C21—C22	−0.5 (3)	C22—C23—O2—C24	−155.38 (15)
C20—C21—C22—C23	0.1 (3)	C25—C24—O2—C23	−177.50 (13)
C19—C18—C23—O2	177.22 (15)	C16—C24—O2—C23	−52.13 (18)
C17—C18—C23—O2	−3.4 (2)	C15—C14—S1—C13	−14.50 (13)
C19—C18—C23—C22	−0.8 (3)	N1—C13—S1—C14	−8.95 (14)
C17—C18—C23—C22	178.54 (15)		

### (I) 6'-Ferrocenyl-6a'-nitro-6',6a',6b',7',9',11a'-hexahydro-2H-spiro[acenaphthylene-1,11'-chromeno[3',4':3,4]pyrrolo[1,2-c]thiazol]-2-one Hydrogen-bond geometry (Å, º)

**Table:**

*D*—H···*A*	*D*—H	H···*A*	*D*···*A*	*D*—H···*A*
C13—H13*A*···O3^i^	0.97	2.50	3.417 (3)	157
C20—H20···O4^ii^	0.93	2.59	3.440 (3)	152
C24—H24···O1	0.98	2.51	3.301 (3)	138

Symmetry codes: (i) *x*+1/2, −*y*+1/2, *z*+1/2; (ii) *x*+1/2, −*y*+1/2, *z*−1/2.

### (II) 6'-(4-Methoxyphenyl)-6a'-nitro-6',6a',6b',7',9',11a'-hexahydro-2H-spiro[acenaphthylene-1,11'-chromeno[3',4':3,4]pyrrolo[1,2-c]thiazol]-2-one Crystal data

**Table:**

C_31_H_24_N_2_O_5_S	*Z* = 2
*M**_r_* = 536.58	*F*(000) = 560
Triclinic, *P*1	*D*_x_ = 1.416 Mg m^−^^3^
*a* = 11.1123 (5) Å	Mo *K*α radiation, λ = 0.71073 Å
*b* = 11.6373 (2) Å	Cell parameters from 5157 reflections
*c* = 12.4095 (3) Å	θ = 2.0–26.4°
α = 117.812 (1)°	µ = 0.18 mm^−^^1^
β = 110.812 (1)°	*T* = 293 K
γ = 95.468 (1)°	Block, colourless
*V* = 1258.89 (7) Å^3^	0.22 × 0.18 × 0.10 mm

### (II) 6'-(4-Methoxyphenyl)-6a'-nitro-6',6a',6b',7',9',11a'-hexahydro-2H-spiro[acenaphthylene-1,11'-chromeno[3',4':3,4]pyrrolo[1,2-c]thiazol]-2-one Data collection

**Table:**

Bruker SMART APEXII area-detector diffractometer	4192 reflections with *I* > 2σ(*I*)
ω and φ scans	*R*_int_ = 0.023
Absorption correction: multi-scan (*SADABS*; Bruker, 2008)	θ_max_ = 26.4°, θ_min_ = 2.0°
*T*_min_ = 0.746, *T*_max_ = 0.845	*h* = −13→13
18670 measured reflections	*k* = −14→14
5157 independent reflections	*l* = −15→15

### (II) 6'-(4-Methoxyphenyl)-6a'-nitro-6',6a',6b',7',9',11a'-hexahydro-2H-spiro[acenaphthylene-1,11'-chromeno[3',4':3,4]pyrrolo[1,2-c]thiazol]-2-one Refinement

**Table:**

Refinement on *F*^2^	0 restraints
Least-squares matrix: full	Hydrogen site location: inferred from neighbouring sites
*R*[*F*^2^ > 2σ(*F*^2^)] = 0.037	H-atom parameters constrained
*wR*(*F*^2^) = 0.105	*w* = 1/[σ^2^(*F*_o_^2^) + (0.0497*P*)^2^ + 0.3546*P*] where *P* = (*F*_o_^2^ + 2*F*_c_^2^)/3
*S* = 1.04	(Δ/σ)_max_ < 0.001
5157 reflections	Δρ_max_ = 0.25 e Å^−^^3^
353 parameters	Δρ_min_ = −0.29 e Å^−^^3^

### (II) 6'-(4-Methoxyphenyl)-6a'-nitro-6',6a',6b',7',9',11a'-hexahydro-2H-spiro[acenaphthylene-1,11'-chromeno[3',4':3,4]pyrrolo[1,2-c]thiazol]-2-one Special details

**Table:**

Geometry. All esds (except the esd in the dihedral angle between two l.s. planes) are estimated using the full covariance matrix. The cell esds are taken into account individually in the estimation of esds in distances, angles and torsion angles; correlations between esds in cell parameters are only used when they are defined by crystal symmetry. An approximate (isotropic) treatment of cell esds is used for estimating esds involving l.s. planes.

### (II) 6'-(4-Methoxyphenyl)-6a'-nitro-6',6a',6b',7',9',11a'-hexahydro-2H-spiro[acenaphthylene-1,11'-chromeno[3',4':3,4]pyrrolo[1,2-c]thiazol]-2-one Fractional atomic coordinates and isotropic or equivalent isotropic displacement parameters (Å^2^)

**Table:**

	*x*	*y*	*z*	*U*_iso_*/*U*_eq_	
C1	0.16234 (14)	0.21087 (14)	1.00050 (14)	0.0303 (3)	
C2	0.29371 (14)	0.31519 (15)	1.06617 (14)	0.0334 (3)	
C3	0.35025 (16)	0.44717 (16)	1.17791 (16)	0.0426 (4)	
H3	0.3036	0.4866	1.2273	0.051*	
C4	0.48101 (18)	0.52074 (18)	1.2151 (2)	0.0555 (5)	
H4	0.5204	0.6108	1.2895	0.067*	
C5	0.55205 (18)	0.46379 (19)	1.1449 (2)	0.0596 (5)	
H5	0.6383	0.5160	1.1729	0.071*	
C6	0.49749 (16)	0.32733 (18)	1.03078 (18)	0.0466 (4)	
C7	0.55832 (18)	0.2539 (2)	0.9491 (2)	0.0587 (5)	
H7	0.6456	0.2956	0.9691	0.070*	
C8	0.48953 (18)	0.1227 (2)	0.8415 (2)	0.0567 (5)	
H8	0.5320	0.0765	0.7898	0.068*	
C9	0.35631 (16)	0.05349 (18)	0.80482 (17)	0.0456 (4)	
H9	0.3117	−0.0359	0.7297	0.055*	
C10	0.29429 (14)	0.12055 (15)	0.88192 (15)	0.0338 (3)	
C11	0.36606 (14)	0.25606 (15)	0.99389 (15)	0.0350 (3)	
C12	0.15158 (13)	0.08333 (14)	0.86708 (14)	0.0296 (3)	
C17	0.04423 (13)	0.07120 (14)	0.73580 (14)	0.0300 (3)	
H17	0.0788	0.0420	0.6679	0.036*	
C18	0.01547 (14)	0.20175 (15)	0.76059 (14)	0.0330 (3)	
C19	0.10115 (16)	0.30340 (17)	0.76795 (17)	0.0422 (4)	
H19	0.1760	0.2885	0.7529	0.051*	
C20	0.07654 (18)	0.42587 (18)	0.7973 (2)	0.0537 (5)	
H20	0.1339	0.4925	0.8008	0.064*	
C21	−0.03321 (18)	0.44951 (18)	0.8216 (2)	0.0539 (5)	
H21	−0.0484	0.5331	0.8437	0.065*	
C22	−0.12025 (16)	0.34983 (16)	0.81319 (17)	0.0464 (4)	
H22	−0.1941	0.3659	0.8300	0.056*	
C23	−0.09723 (14)	0.22551 (15)	0.77958 (15)	0.0358 (3)	
C24	−0.17388 (14)	0.00433 (14)	0.75027 (14)	0.0326 (3)	
H24	−0.1306	0.0227	0.8431	0.039*	
C25	−0.31406 (14)	−0.09614 (14)	0.67748 (14)	0.0320 (3)	
C30	−0.41836 (15)	−0.10188 (16)	0.56946 (16)	0.0396 (3)	
H30	−0.4010	−0.0438	0.5410	0.047*	
C29	−0.54640 (16)	−0.19207 (17)	0.50438 (17)	0.0453 (4)	
H29	−0.6151	−0.1937	0.4332	0.054*	
C28	−0.57380 (15)	−0.28045 (17)	0.54407 (16)	0.0426 (4)	
C31	−0.7326 (2)	−0.4772 (2)	0.4878 (2)	0.0693 (6)	
H31A	−0.6753	−0.5323	0.4670	0.104*	
H31B	−0.8259	−0.5322	0.4271	0.104*	
H31C	−0.7160	−0.4419	0.5806	0.104*	
C27	−0.47286 (17)	−0.27538 (18)	0.65139 (18)	0.0481 (4)	
H27	−0.4906	−0.3335	0.6797	0.058*	
C26	−0.34419 (15)	−0.18291 (18)	0.71723 (16)	0.0420 (4)	
H26	−0.2765	−0.1795	0.7902	0.050*	
C16	−0.07894 (14)	−0.04652 (14)	0.68327 (13)	0.0313 (3)	
C15	−0.01600 (15)	−0.14060 (14)	0.72294 (15)	0.0355 (3)	
H15	−0.0812	−0.1964	0.7299	0.043*	
C14	0.04383 (17)	−0.23249 (16)	0.63316 (17)	0.0456 (4)	
H14A	0.0794	−0.1880	0.5964	0.055*	
H14B	−0.0246	−0.3194	0.5579	0.055*	
C13	0.18494 (18)	−0.11019 (17)	0.90177 (18)	0.0451 (4)	
H13A	0.1559	−0.1419	0.9510	0.054*	
H13B	0.2769	−0.0476	0.9621	0.054*	
N1	0.09591 (12)	−0.04224 (12)	0.85801 (12)	0.0338 (3)	
N2	−0.16219 (13)	−0.11755 (14)	0.53086 (13)	0.0409 (3)	
O1	0.07738 (10)	0.21597 (11)	1.04066 (10)	0.0397 (3)	
O2	−0.19534 (11)	0.12778 (11)	0.76151 (12)	0.0450 (3)	
O3	−0.17443 (13)	−0.04707 (14)	0.48225 (12)	0.0574 (3)	
O4	−0.21819 (13)	−0.24098 (12)	0.46534 (12)	0.0571 (3)	
O5	−0.70369 (12)	−0.36725 (14)	0.47129 (14)	0.0640 (4)	
S1	0.17845 (6)	−0.25726 (5)	0.74712 (6)	0.06168 (16)	

### (II) 6'-(4-Methoxyphenyl)-6a'-nitro-6',6a',6b',7',9',11a'-hexahydro-2H-spiro[acenaphthylene-1,11'-chromeno[3',4':3,4]pyrrolo[1,2-c]thiazol]-2-one Atomic displacement parameters (Å^2^)

**Table:**

	*U*^11^	*U*^22^	*U*^33^	*U*^12^	*U*^13^	*U*^23^
C1	0.0301 (7)	0.0321 (7)	0.0293 (7)	0.0130 (6)	0.0119 (6)	0.0175 (6)
C2	0.0312 (7)	0.0345 (8)	0.0328 (7)	0.0120 (6)	0.0114 (6)	0.0187 (6)
C3	0.0398 (8)	0.0347 (8)	0.0419 (8)	0.0123 (7)	0.0141 (7)	0.0157 (7)
C4	0.0441 (9)	0.0352 (9)	0.0585 (11)	0.0029 (7)	0.0138 (8)	0.0135 (8)
C5	0.0361 (9)	0.0491 (11)	0.0702 (12)	−0.0016 (8)	0.0169 (8)	0.0235 (10)
C6	0.0313 (8)	0.0496 (10)	0.0552 (10)	0.0094 (7)	0.0171 (7)	0.0280 (8)
C7	0.0335 (9)	0.0705 (13)	0.0716 (12)	0.0135 (9)	0.0282 (9)	0.0353 (11)
C8	0.0428 (9)	0.0705 (13)	0.0626 (11)	0.0248 (9)	0.0348 (9)	0.0309 (10)
C9	0.0397 (8)	0.0479 (10)	0.0469 (9)	0.0175 (7)	0.0242 (7)	0.0198 (8)
C10	0.0295 (7)	0.0388 (8)	0.0360 (7)	0.0137 (6)	0.0151 (6)	0.0215 (7)
C11	0.0291 (7)	0.0377 (8)	0.0383 (8)	0.0115 (6)	0.0133 (6)	0.0219 (7)
C12	0.0293 (7)	0.0296 (7)	0.0312 (7)	0.0114 (6)	0.0147 (6)	0.0162 (6)
C17	0.0293 (7)	0.0315 (7)	0.0285 (7)	0.0087 (6)	0.0140 (5)	0.0153 (6)
C18	0.0320 (7)	0.0331 (7)	0.0294 (7)	0.0081 (6)	0.0090 (6)	0.0175 (6)
C19	0.0386 (8)	0.0416 (9)	0.0494 (9)	0.0092 (7)	0.0171 (7)	0.0292 (8)
C20	0.0474 (10)	0.0427 (10)	0.0685 (12)	0.0070 (8)	0.0164 (9)	0.0366 (9)
C21	0.0479 (10)	0.0341 (9)	0.0647 (11)	0.0123 (7)	0.0108 (8)	0.0266 (8)
C22	0.0367 (8)	0.0380 (9)	0.0515 (9)	0.0139 (7)	0.0124 (7)	0.0200 (8)
C23	0.0308 (7)	0.0315 (7)	0.0349 (7)	0.0068 (6)	0.0088 (6)	0.0157 (6)
C24	0.0317 (7)	0.0324 (7)	0.0315 (7)	0.0098 (6)	0.0152 (6)	0.0151 (6)
C25	0.0299 (7)	0.0331 (7)	0.0316 (7)	0.0105 (6)	0.0142 (6)	0.0162 (6)
C30	0.0368 (8)	0.0396 (8)	0.0413 (8)	0.0091 (7)	0.0123 (7)	0.0255 (7)
C29	0.0347 (8)	0.0464 (9)	0.0422 (9)	0.0078 (7)	0.0054 (7)	0.0245 (8)
C28	0.0327 (8)	0.0390 (8)	0.0441 (9)	0.0042 (6)	0.0141 (7)	0.0176 (7)
C31	0.0636 (12)	0.0463 (11)	0.0810 (14)	−0.0031 (9)	0.0374 (11)	0.0227 (10)
C27	0.0418 (9)	0.0533 (10)	0.0589 (10)	0.0097 (8)	0.0215 (8)	0.0389 (9)
C26	0.0352 (8)	0.0543 (10)	0.0431 (8)	0.0129 (7)	0.0145 (7)	0.0334 (8)
C16	0.0310 (7)	0.0304 (7)	0.0269 (7)	0.0069 (6)	0.0132 (6)	0.0118 (6)
C15	0.0376 (8)	0.0282 (7)	0.0392 (8)	0.0090 (6)	0.0203 (6)	0.0151 (6)
C14	0.0523 (10)	0.0332 (8)	0.0487 (9)	0.0159 (7)	0.0277 (8)	0.0163 (7)
C13	0.0530 (10)	0.0438 (9)	0.0514 (9)	0.0249 (8)	0.0262 (8)	0.0311 (8)
N1	0.0363 (6)	0.0312 (6)	0.0373 (6)	0.0129 (5)	0.0174 (5)	0.0198 (5)
N2	0.0362 (7)	0.0433 (8)	0.0306 (6)	0.0059 (6)	0.0147 (5)	0.0126 (6)
O1	0.0362 (5)	0.0429 (6)	0.0366 (6)	0.0130 (5)	0.0201 (5)	0.0162 (5)
O2	0.0356 (6)	0.0318 (6)	0.0648 (7)	0.0117 (5)	0.0261 (5)	0.0214 (5)
O3	0.0608 (8)	0.0663 (8)	0.0370 (6)	0.0120 (6)	0.0136 (6)	0.0296 (6)
O4	0.0537 (7)	0.0416 (7)	0.0383 (6)	−0.0039 (6)	0.0137 (5)	0.0034 (5)
O5	0.0394 (6)	0.0582 (8)	0.0674 (8)	−0.0082 (6)	0.0094 (6)	0.0297 (7)
S1	0.0781 (4)	0.0513 (3)	0.0716 (3)	0.0425 (3)	0.0426 (3)	0.0342 (3)

### (II) 6'-(4-Methoxyphenyl)-6a'-nitro-6',6a',6b',7',9',11a'-hexahydro-2H-spiro[acenaphthylene-1,11'-chromeno[3',4':3,4]pyrrolo[1,2-c]thiazol]-2-one Geometric parameters (Å, º)

**Table:**

C1—O1	1.2085 (17)	C22—H22	0.9300
C1—C2	1.471 (2)	C23—O2	1.3728 (18)
C1—C12	1.5783 (19)	C24—O2	1.4291 (18)
C2—C3	1.373 (2)	C24—C25	1.5090 (19)
C2—C11	1.402 (2)	C24—C16	1.5612 (19)
C3—C4	1.405 (2)	C24—H24	0.9800
C3—H3	0.9300	C25—C26	1.376 (2)
C4—C5	1.367 (3)	C25—C30	1.394 (2)
C4—H4	0.9300	C30—C29	1.373 (2)
C5—C6	1.415 (3)	C30—H30	0.9300
C5—H5	0.9300	C29—C28	1.383 (2)
C6—C11	1.404 (2)	C29—H29	0.9300
C6—C7	1.415 (3)	C28—O5	1.3652 (18)
C7—C8	1.359 (3)	C28—C27	1.374 (2)
C7—H7	0.9300	C31—O5	1.413 (2)
C8—C9	1.414 (2)	C31—H31A	0.9600
C8—H8	0.9300	C31—H31B	0.9600
C9—C10	1.369 (2)	C31—H31C	0.9600
C9—H9	0.9300	C27—C26	1.388 (2)
C10—C11	1.408 (2)	C27—H27	0.9300
C10—C12	1.5242 (19)	C26—H26	0.9300
C12—N1	1.4682 (18)	C16—N2	1.5061 (18)
C12—C17	1.5714 (19)	C16—C15	1.528 (2)
C17—C18	1.494 (2)	C15—N1	1.4572 (19)
C17—C16	1.5331 (19)	C15—C14	1.542 (2)
C17—H17	0.9800	C15—H15	0.9800
C18—C23	1.384 (2)	C14—S1	1.8111 (18)
C18—C19	1.392 (2)	C14—H14A	0.9700
C19—C20	1.378 (2)	C14—H14B	0.9700
C19—H19	0.9300	C13—N1	1.438 (2)
C20—C21	1.379 (3)	C13—S1	1.8521 (17)
C20—H20	0.9300	C13—H13A	0.9700
C21—C22	1.376 (2)	C13—H13B	0.9700
C21—H21	0.9300	N2—O3	1.2164 (18)
C22—C23	1.384 (2)	N2—O4	1.2215 (17)
			
O1—C1—C2	127.79 (13)	O2—C24—C25	105.19 (11)
O1—C1—C12	124.36 (12)	O2—C24—C16	113.86 (11)
C2—C1—C12	107.80 (11)	C25—C24—C16	114.99 (11)
C3—C2—C11	120.59 (14)	O2—C24—H24	107.5
C3—C2—C1	132.13 (14)	C25—C24—H24	107.5
C11—C2—C1	107.27 (12)	C16—C24—H24	107.5
C2—C3—C4	117.80 (16)	C26—C25—C30	117.72 (13)
C2—C3—H3	121.1	C26—C25—C24	121.29 (13)
C4—C3—H3	121.1	C30—C25—C24	120.97 (13)
C5—C4—C3	121.91 (16)	C29—C30—C25	120.97 (14)
C5—C4—H4	119.0	C29—C30—H30	119.5
C3—C4—H4	119.0	C25—C30—H30	119.5
C4—C5—C6	121.62 (16)	C30—C29—C28	120.37 (15)
C4—C5—H5	119.2	C30—C29—H29	119.8
C6—C5—H5	119.2	C28—C29—H29	119.8
C11—C6—C5	115.69 (16)	O5—C28—C27	124.78 (15)
C11—C6—C7	116.01 (16)	O5—C28—C29	115.62 (15)
C5—C6—C7	128.30 (16)	C27—C28—C29	119.60 (14)
C8—C7—C6	120.19 (16)	O5—C31—H31A	109.5
C8—C7—H7	119.9	O5—C31—H31B	109.5
C6—C7—H7	119.9	H31A—C31—H31B	109.5
C7—C8—C9	122.90 (16)	O5—C31—H31C	109.5
C7—C8—H8	118.6	H31A—C31—H31C	109.5
C9—C8—H8	118.6	H31B—C31—H31C	109.5
C10—C9—C8	118.72 (16)	C28—C27—C26	119.50 (15)
C10—C9—H9	120.6	C28—C27—H27	120.2
C8—C9—H9	120.6	C26—C27—H27	120.2
C9—C10—C11	118.31 (14)	C25—C26—C27	121.81 (14)
C9—C10—C12	132.99 (14)	C25—C26—H26	119.1
C11—C10—C12	108.51 (12)	C27—C26—H26	119.1
C2—C11—C6	122.37 (14)	N2—C16—C15	111.92 (12)
C2—C11—C10	113.76 (13)	N2—C16—C17	110.50 (11)
C6—C11—C10	123.87 (14)	C15—C16—C17	103.66 (11)
N1—C12—C10	120.51 (12)	N2—C16—C24	107.57 (11)
N1—C12—C17	104.32 (11)	C15—C16—C24	111.11 (11)
C10—C12—C17	110.66 (11)	C17—C16—C24	112.14 (11)
N1—C12—C1	108.87 (11)	N1—C15—C16	101.78 (11)
C10—C12—C1	101.87 (11)	N1—C15—C14	107.69 (12)
C17—C12—C1	110.56 (11)	C16—C15—C14	116.88 (13)
C18—C17—C16	114.61 (12)	N1—C15—H15	110.0
C18—C17—C12	114.00 (11)	C16—C15—H15	110.0
C16—C17—C12	103.47 (11)	C14—C15—H15	110.0
C18—C17—H17	108.1	C15—C14—S1	104.77 (11)
C16—C17—H17	108.1	C15—C14—H14A	110.8
C12—C17—H17	108.1	S1—C14—H14A	110.8
C23—C18—C19	118.15 (14)	C15—C14—H14B	110.8
C23—C18—C17	120.18 (13)	S1—C14—H14B	110.8
C19—C18—C17	121.66 (14)	H14A—C14—H14B	108.9
C20—C19—C18	120.87 (16)	N1—C13—S1	107.70 (11)
C20—C19—H19	119.6	N1—C13—H13A	110.2
C18—C19—H19	119.6	S1—C13—H13A	110.2
C19—C20—C21	119.93 (16)	N1—C13—H13B	110.2
C19—C20—H20	120.0	S1—C13—H13B	110.2
C21—C20—H20	120.0	H13A—C13—H13B	108.5
C22—C21—C20	120.20 (16)	C13—N1—C15	110.12 (12)
C22—C21—H21	119.9	C13—N1—C12	120.18 (12)
C20—C21—H21	119.9	C15—N1—C12	111.07 (11)
C21—C22—C23	119.54 (16)	O3—N2—O4	124.37 (14)
C21—C22—H22	120.2	O3—N2—C16	117.71 (13)
C23—C22—H22	120.2	O4—N2—C16	117.82 (13)
O2—C23—C18	123.07 (13)	C23—O2—C24	121.41 (11)
O2—C23—C22	115.68 (14)	C28—O5—C31	118.60 (15)
C18—C23—C22	121.20 (14)	C14—S1—C13	92.72 (7)
			
O1—C1—C2—C3	−7.6 (3)	O2—C24—C25—C26	−138.70 (14)
C12—C1—C2—C3	174.87 (16)	C16—C24—C25—C26	95.21 (17)
O1—C1—C2—C11	171.08 (14)	O2—C24—C25—C30	39.81 (17)
C12—C1—C2—C11	−6.50 (15)	C16—C24—C25—C30	−86.28 (17)
C11—C2—C3—C4	1.2 (2)	C26—C25—C30—C29	−0.6 (2)
C1—C2—C3—C4	179.63 (16)	C24—C25—C30—C29	−179.14 (14)
C2—C3—C4—C5	−1.1 (3)	C25—C30—C29—C28	−0.8 (3)
C3—C4—C5—C6	0.0 (3)	C30—C29—C28—O5	−179.01 (16)
C4—C5—C6—C11	0.9 (3)	C30—C29—C28—C27	1.5 (3)
C4—C5—C6—C7	−179.3 (2)	O5—C28—C27—C26	179.69 (16)
C11—C6—C7—C8	0.5 (3)	C29—C28—C27—C26	−0.9 (3)
C5—C6—C7—C8	−179.3 (2)	C30—C25—C26—C27	1.2 (2)
C6—C7—C8—C9	0.5 (3)	C24—C25—C26—C27	179.76 (15)
C7—C8—C9—C10	−1.0 (3)	C28—C27—C26—C25	−0.5 (3)
C8—C9—C10—C11	0.5 (2)	C18—C17—C16—N2	82.79 (14)
C8—C9—C10—C12	174.67 (16)	C12—C17—C16—N2	−152.48 (11)
C3—C2—C11—C6	−0.2 (2)	C18—C17—C16—C15	−157.15 (11)
C1—C2—C11—C6	−179.06 (14)	C12—C17—C16—C15	−32.42 (13)
C3—C2—C11—C10	−179.84 (14)	C18—C17—C16—C24	−37.21 (16)
C1—C2—C11—C10	1.34 (17)	C12—C17—C16—C24	87.52 (13)
C5—C6—C11—C2	−0.8 (2)	O2—C24—C16—N2	−79.34 (14)
C7—C6—C11—C2	179.40 (16)	C25—C24—C16—N2	42.14 (16)
C5—C6—C11—C10	178.78 (16)	O2—C24—C16—C15	157.84 (11)
C7—C6—C11—C10	−1.0 (2)	C25—C24—C16—C15	−80.67 (15)
C9—C10—C11—C2	−179.85 (14)	O2—C24—C16—C17	42.35 (16)
C12—C10—C11—C2	4.61 (17)	C25—C24—C16—C17	163.83 (12)
C9—C10—C11—C6	0.6 (2)	N2—C16—C15—N1	158.74 (11)
C12—C10—C11—C6	−174.99 (14)	C17—C16—C15—N1	39.65 (13)
C9—C10—C12—N1	56.9 (2)	C24—C16—C15—N1	−80.99 (13)
C11—C10—C12—N1	−128.44 (13)	N2—C16—C15—C14	41.75 (17)
C9—C10—C12—C17	−65.0 (2)	C17—C16—C15—C14	−77.34 (15)
C11—C10—C12—C17	109.62 (13)	C24—C16—C15—C14	162.02 (12)
C9—C10—C12—C1	177.43 (17)	N1—C15—C14—S1	37.43 (14)
C11—C10—C12—C1	−7.94 (15)	C16—C15—C14—S1	151.13 (11)
O1—C1—C12—N1	−40.63 (18)	S1—C13—N1—C15	30.38 (15)
C2—C1—C12—N1	137.05 (11)	S1—C13—N1—C12	−100.60 (13)
O1—C1—C12—C10	−168.95 (13)	C16—C15—N1—C13	−168.37 (12)
C2—C1—C12—C10	8.73 (14)	C14—C15—N1—C13	−44.91 (16)
O1—C1—C12—C17	73.41 (17)	C16—C15—N1—C12	−32.75 (14)
C2—C1—C12—C17	−108.91 (12)	C14—C15—N1—C12	90.71 (14)
N1—C12—C17—C18	138.20 (12)	C10—C12—N1—C13	18.04 (19)
C10—C12—C17—C18	−90.79 (14)	C17—C12—N1—C13	143.01 (13)
C1—C12—C17—C18	21.31 (16)	C1—C12—N1—C13	−98.94 (14)
N1—C12—C17—C16	13.06 (13)	C10—C12—N1—C15	−112.52 (14)
C10—C12—C17—C16	144.08 (11)	C17—C12—N1—C15	12.44 (14)
C1—C12—C17—C16	−103.82 (12)	C1—C12—N1—C15	130.49 (12)
C16—C17—C18—C23	20.89 (18)	C15—C16—N2—O3	−152.92 (13)
C12—C17—C18—C23	−98.09 (15)	C17—C16—N2—O3	−37.95 (17)
C16—C17—C18—C19	−160.37 (13)	C24—C16—N2—O3	84.76 (15)
C12—C17—C18—C19	80.65 (17)	C15—C16—N2—O4	30.56 (18)
C23—C18—C19—C20	1.9 (2)	C17—C16—N2—O4	145.53 (13)
C17—C18—C19—C20	−176.89 (15)	C24—C16—N2—O4	−91.76 (15)
C18—C19—C20—C21	0.8 (3)	C18—C23—O2—C24	13.6 (2)
C19—C20—C21—C22	−1.7 (3)	C22—C23—O2—C24	−168.94 (13)
C20—C21—C22—C23	−0.3 (3)	C25—C24—O2—C23	−157.98 (12)
C19—C18—C23—O2	173.50 (14)	C16—C24—O2—C23	−31.20 (18)
C17—C18—C23—O2	−7.7 (2)	C27—C28—O5—C31	−12.7 (3)
C19—C18—C23—C22	−3.9 (2)	C29—C28—O5—C31	167.94 (17)
C17—C18—C23—C22	174.92 (14)	C15—C14—S1—C13	−17.28 (12)
C21—C22—C23—O2	−174.43 (15)	N1—C13—S1—C14	−6.37 (12)
C21—C22—C23—C18	3.1 (2)		

### (II) 6'-(4-Methoxyphenyl)-6a'-nitro-6',6a',6b',7',9',11a'-hexahydro-2H-spiro[acenaphthylene-1,11'-chromeno[3',4':3,4]pyrrolo[1,2-c]thiazol]-2-one Hydrogen-bond geometry (Å, º)

Cg1 and Cg2 are the centroids of the C25–C30 and C2–C11 rings, respectively.

**Table:**

*D*—H···*A*	*D*—H	H···*A*	*D*···*A*	*D*—H···*A*
C17—H17···O3^i^	0.98	2.47	3.412 (2)	161
C24—H24···O1	0.98	2.50	3.178 (19)	126
C8—H8···*Cg*1^ii^	0.93	2.82	3.759 (2)	148
C27—H27···*Cg*2^iii^	0.93	2.79	3.720 (3)	149

Symmetry codes: (i) −*x*, −*y*, −*z*+1; (ii) *x*+1, *y*, *z*; (iii) −*x*, −*y*, −*z*+2.

## References

[bb1] Bruker (2008). *APEX2*, *SAINT* and *SADABS*. Bruker AXS Inc., Madison, Wisconsin, USA.

[bb2] Čačić, M., Molnar, M., Šarkanj, B., Has-Schön, E. & Rajković, V. (2010). *Molecules*, **15**, 6795–6809. 10.3390/molecules15106795 PMC6259306 20881932

[bb3] Farrugia, L. J. (2012). *J. Appl. Cryst.* **45**, 849–854.

[bb4] Fun, H.-K., Hemamalini, M., Shanmugavelan, P., Ponnuswamy, A. & Jagatheesan, R. (2011). *Acta Cryst.* E**67**, o2706. 10.1107/S1600536811037706 PMC3201390 22064819

[bb5] Majed, A. A. & Abid, D. S. (2015). *Basrah J. Sci.* **33**, 101–117.

[bb6] Pandey, Y., Sharma, P. K., Kumar, N. & Singh, A. (2011). *Int. J. Pharm. Tech Res.* **3**, 980–985.

[bb7] Samadhiya, P., Sharma, R., Srivastava, S. K. & Srivastava, S. D. (2012). *Leonardo J. Sci.* **20**, 37–58.

[bb8] Sheldrick, G. M. (2008). *Acta Cryst.* A**64**, 112–122. 10.1107/S0108767307043930 18156677

[bb9] Sheldrick, G. M. (2015). *Acta Cryst.* C**71**, 3–8.

[bb10] Shih, M. H., Xu, Y. Y., Yang, Y. S. & Lin, G. L. (2015). *Molecules*, **20**, 6520–6532. 10.3390/molecules20046520 PMC6272598 25871371

[bb11] Spek, A. L. (2009). *Acta Cryst.* D**65**, 148–155. 10.1107/S090744490804362X PMC2631630 19171970

[bb12] Vennila, J. P., Thiruvadigal, D. J., Kavitha, H. P., Chakkaravarthi, G. & Manivannan, V. (2011). *Acta Cryst.* E**67**, o1902. 10.1107/S1600536811025281 PMC3212296 22090953

